# Seroprevalence and risk factors of anisakiasis associated with raw seafood consumption in the Sangihe Islands, Indonesia

**DOI:** 10.5455/javar.2025.l867

**Published:** 2025-03-23

**Authors:** Dhito Dwi Pramardika, Fadjar Satrija, Sulistiono Sulistiono, Risa Tiuria, Arifin Budiman Nugraha, Sri Murtini

**Affiliations:** 1 Veterinary Biomedical Sciences Study Program, Graduate School of IPB University, Bogor, 16680, Indonesia; 2 Department of Health, Nusa Utara State Polytechnic, Sangihe Islands, North Sulawesi, 95812, Indonesia; 3 Division of Parasitology and Medical Entomology, School of Veterinary Medicine and Biomedical Sciences, IPB University, Bogor, 16680, Indonesia; 4 Department of Aquatic Resources Management, Faculty of Fisheries and Marine Sciences, IPB University, Bogor, 16680, Indonesia; 5 Division of Medical Microbiology, School of Veterinary Medicine and Biomedical Sciences, IPB University, Bogor, 16680, Indonesia

**Keywords:** Foodborne parasitic infection, *Katsuwonus pelamis*, raw fish consumption, seroprevalence, zoonosis

## Abstract

**Objective:**

This study aimed to determine the seroprevalence of anisakiasis and its associated risk factors in the Sangihe Islands Regency, Indonesia, where people have a unique habit of consuming raw seafood “Kinilo.”

**Materials and Methods:**

This cross-sectional study involved 112 respondents who met the inclusion criteria using the Indirect ELISA method with the *Anisakidae* immunoglobulin G (IgG) kit.

**Results:**

The results showed that 59/112 respondents (52.67%) were seropositive for Anisakidae IgG, with significant risk factors such as the consumption of *Katsuwonus pelamis* and measures related to eating habits. Multivariate analysis revealed that consumption of raw *K. pelamis* was associated with a 45.748 times higher risk of anisakiasis.

**Conclusion:**

These findings highlight the need to raise awareness and implement interventions to prevent infection, including fish processing and storage education. This study emphasizes the need for a holistic health approach to reduce the risk factors for anisakiasis in at-risk communities.

## Introduction

Anisakiasis is caused by consuming raw or undercooked fish infected with ascaridoid nematodes of the Anisakidae family, including *Anisakis* sp., *Pseudoterranova decipiens*, and *Contracaecum* sp. Symptoms include acute abdominal pain that usually occurs within hours of larval ingestion. This non-specific abdominal distress can be mistaken for other conditions such as peptic ulcers, food poisoning, and appendicitis. If the larvae enter the intestine, a severe eosinophilic granulomatous response may also occur 1 to 2 weeks after infection, causing symptoms resembling Crohn’s disease, although intestinal perforation may occur, although this is rare [1]. Additionally, anisakiasis may increase the risk of gastric or colon adenocarcinoma [2].

Globally, anisakiasis has been reported in 20,000 cases, with > 90% occurring in Japan, which has a habit of eating raw fish such as sashimi [3,4]. In the period 1971–2022, Korea reported 851 cases of anisakiasis [5]. In Southeast Asia, the first case was reported in Thailand in 1993, followed by Malaysia in Sarawak and Borneo [6,7]. In Indonesia, the only report showed that 11% of the respondents in Sidoarjo were seropositive for *Anisakis* spp. [8].

Recent studies from neighboring countries have indicated the presence of *Anisakis* larvae in various fish species. Studies in the Philippines reported *Anisakis* sp. infection in *Decapterus tabl* (27.69%), *D. macrosoma* (19%), and *D. maruadsi* (17.50%) [9]. A study in Malaysia reported 100% infection with *Anisakis typica* in *Decapterus macrosoma* [10]. Additionally, studies in Indonesia identified six marine fish families that are most frequently infected with *Anisakis* sp.: Balistidae, Carangidae, Epinephelidae, Lutjanidae, Priacanthidae, and Scombridae [11]. These findings suggest a potential risk of anisakiasis in Indonesia.

Risk factors that cause anisakiasis in humans include the consumption of raw fish, fish with vinegar, and smoked fish [12]. Another study also reported that men over 45 years of age and those who consume alcohol are at risk of anisakiasis [13]. Although the risk factors for anisakiasis have been widely studied, studies in Indonesia are lacking in identifying the specific fish species consumed that may contribute to anisakiasis.

The Sangihe Islands Regency is a regency in Indonesia that borders the southern Philippines. People in this district have a unique eating habit called “Kinilo” (the consumption of raw seafood with lemon juice). Kinilo is a specialty food of the Sangihe community that not only serves as a meal but also has deep cultural value. It is often consumed in daily life, especially by adult males, and is a mandatory dish for family events and social gatherings. Its processing, which involves cutting fresh fish without heating or freezing, increases the risk of parasitic infections, especially from *Anisakis spp*. In Kinilo preparations, various types of fish are used as the main ingredients, including *Katsuwonus pelamis*,* Hemiramphus brasiliensis*,* Euthynnus affinis*, *Thunnus albacares*, and several other types of fish. Similar practices are found in various parts of the world, such as sashimi in Japan and ceviche in Latin America; however, Kinilo is unique in its use of lemons as the only processing method. Unfortunately, the lack of public knowledge regarding the risk of parasites in raw fish may increase the likelihood of anisakiasis, which could impact public health in the region. To date, no anisakiasis cases have been reported in this district.

This study aimed to determine the seroprevalence of anisakiasis in the Sangihe Island Regency, identify the risk factors associated with anisakiasis, and determine the most dominant factor contributing to the incidence of anisakiasis. In addition, this study is expected to generate significant new data on the prevalence and risk factors of anisakiasis in this region, which has not been previously documented. This study is also expected to provide important information for the community and authorities, with a focus on health impacts, to reduce the risk of anisakiasis in the Indonesian Archipelago region where raw seafood is commonly eaten.

## Materials and Methods

### Ethical approval

The present study protocol was reviewed and approved by the Health Research Ethics Committee of the Manado Health Polytechnic, Ministry of Health, Indonesia (approval No. KEPK.01/12/464/2023) on 19 December 2023. Informed consent was submitted by all subjects when they were enrolled.

### Study design and sampling

This study was a cross-sectional study. This study was conducted from January to May 2024 in Sangihe Islands Regency. The sampling technique employed was percentage estimation using the WinEpi software, last updated in 2010 (http://www.winepi.net/uk/index.htm) [14]. The confidence interval value used was 95%, the prevalence was 7% [15], and the error value was 5%. A total of 101 respondents were obtained. To avoid the dropout of respondents, the sample size was increased by 10% so that the total sample was 112 respondents.

The research sample included people living in the Sangihe Islands Regency with the following inclusion criteria: consuming raw seafood with lemon juice “Kinilo” aged ≥ 17 years and having good physical and mental health. Exclusion criteria are pregnant women and respondents who were on certain medications, especially those taking immunosuppressants or corticosteroids. The sampling method used was voluntary response sampling, which provides information on the willingness to participate in research through social media (WhatsApp and Facebook). Data and blood samples were collected from the respondents who consented to be part of the study after being contacted and scheduled for a visit.

### Data collection and study variables

Respondents, who met the inclusion and exclusion criteria, were interviewed, and data were collected using a questionnaire. The data included sociodemographic information (age, gender, address, ethnicity, religion, marital status, education, occupation, income, and raw seafood “Kinilo,” which was frequently consumed). Interviews were also conducted regarding common symptoms of anisakiasis (abdominal pain, nausea, vomiting, and diarrhea).

The total number of knowledge, attitude, and practice questions was 26. The questions were tested for validity (r > r table) and reliability (Cronbach’s *α* > 0.6). The indices of knowledge, attitude, and practice assessment results were then categorized into several categories to facilitate the analysis. The knowledge assessment index was categorized into three categories: good (> 76%), fair (60%–75%), and poor (< 60%). The attitude category was grouped as positive (≥ 66.7%) and negative (< 66.7%). The practice assessment index was grouped into good (≥ 50%) and poor practice (< 50%).

Respondents provided a blood sample with a volume of 1.5 cc, following the established blood collection protocol [16]. Blood samples were then stored in a yellow lid vacutainer and centrifuged for 10 min at 1.575 G. The separated serum was then transferred into a 1.5 ml microtube, followed by inactivation using a water bath at 56°C for 30 min, and stored in a freezer at −20°C until serological examination.

Detecting immunoglobulin G (IgG) antibodies against Anisakidae in human serum is essential for assessing health risks. Using the Anisakidae IgG ELISA kit from Bordier Affinity Products, Switzerland, we can accurately identify these antibodies through an indirect ELISA procedure. Begin by adding a dilution buffer to all wells and incubating for 5 to 15 min at ambient temperature for blocking. After the incubation, the buffer was removed, and the first well with 100 µl of dilution buffer for the no-serum blank. The following three wells should contain 100 µl each of diluted negative control, weak positive control (cut-off), and positive control serum. For those using more than 25 samples, the controls in the last three wells were used for duplication. Filled the remaining with 100 µl of diluted samples. The wells are then covered with adhesive tape and incubated for 30 min at 37°C. After removing the sera, the wells were washed four times with about 250 µl of washing solution. Then, 100 µl of the diluted conjugate was added to each well, the plate was covered, and incubated for 30 min at 37°C. After incubation then, the conjugate was removed, and the wells were washed four more times with the washing solution. The substrate solution was then added to all wells, as much as 100 µl, and incubated for another 30 min at 37°C. To stop the reaction, 100 µl was added to each well. Finally, the absorbances were measured at 405 nm within 1 h to obtain precise results [17].

### Statistical analysis

The collected data were analyzed using univariate, bivariate, and multivariate analyses. The Spearman rank test was used for bivariate analysis because the data obtained were not normally distributed. The strength of the correlation follows the absolute value guidelines: weak (*r* < 0.40), moderate (*r* = 0.40 to 0.69), and strong (*r* ≥ 0.70) [18]. Multivariate analysis was used to determine the risk factor value or odds ratio (OR) of the independent variables on the dependent variable using multiple logistic regression models. All analyses were performed using SPSS software version 25 (IBM, Chicago, IL, USA).

## Results

### Sociodemographic characteristics and prevalence of anisakiasis risk factors

This study involved 112 respondents from the Sangihe Islands Regency. Most participants were aged 25 to 34 years (31/112 respondents, 27.6%), male (74/112 respondents, 66.1%), and residing in the North Tabukan sub-district (80/112 respondents, 73.2%). The majority were of Sangihe ethnicity (109/112 respondents, 97.3%), Christian Protestants (65/112 respondents, 58%), married (75/112 respondents, 67%), had a high school education (58/112 respondents, 51.7%), worked as housewives (26/112 respondents, 23.2%), and earned < Rp. 971,488 (82/112 respondents, 73.3%).

Regarding anisakiasis-related knowledge, 109/112 respondents (97.3%) had insufficient knowledge about the disease. However, 68/112 respondents (60.7%) exhibited positive attitudes such as seeking medical help if they experienced symptoms after eating raw fish, freezing important fish before processing, and being more careful in choosing fish. Meanwhile, 80/112 respondents (71.4%) had poor practices such as improper fish cleaning, cooking fish with offal, and feeding fish offal to livestock (Table 1). Despite this, all the respondents reported being in good health without symptoms.

### The seroprevalence of Anisakiasis and associated factors

The ELISA results showed that 59/112 respondents (52.67%) were seropositive for Anisakidae antigens (Table 1). The bivariate tests showed that the variables that correlated with seropositive anisakiasis were the respondents’ residential area* (p* = 0.009), raw fish consumption (*p* = 0.003), and practice variables (*p* = 0.003). Based on the level of correlation strength, the respondents’ residential area variable had a weak correlation (*r* = 0.246), the raw fish consumption variable was weakly correlated (*r* = 0.281), and the practice variable (*r* = 0.283) was also weakly correlated (Table 1).

**Figure 1.**
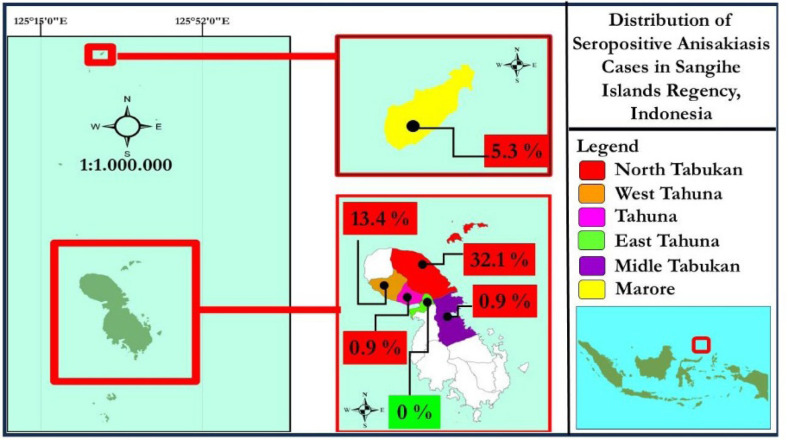
Distribution of seropositive anisakiasis cases in Sangihe Islands Regency, Indonesia.

Overall, based on the number of samples in the Sangihe Islands Regency, 36/112 respondents (32.1%) living in the North Tabukan sub-district showed a seropositive IgG reaction to anisakiasis (Fig. 1). Another interesting finding was that the raw fish that were consumed and experienced anisakiasis were *H. brasiliensis* (15.2%) and *K. pelamis* (14.3%). The results also indicated that the practice was poor, and the patient had anisakiasis (31.3%) (Table 1).

### Dominant factors associated with anisakiasis

Multivariate logistic regression analysis was used to identify the most important variables associated with anisakiasis. Eight of the candidate variables satisfied the inclusion criteria (*p *> 0.25) and were added to the model. After nine iterations of model refinement, the final model maintained variables with a *p*-value < 0.05.

**Table 1.** Factors correlated with anisakiasis.

Variable	Anisakiasis				Total (%)	*r*	*p *value
	Seronegative (*n* = 53)		Seropositive (*n* = 59)				
	*N*	%	*N*	%			
**Age (years)**						0.137	0.151
17–24	10	9.0	10	9.0	20 (17.9)		
25–34	19	17.0	12	10.7	31 (27.6)		
35–44	11	9.8	14	12.5	25 (22.3)		
45–54	4	3.5	12	10.7	16 (14.3)		
≥ 55	9	8.0	11	9.8	20 (17.9)		
**Sex**						–0.038	0.687
Male	34	30.3	40	35.7	74 (66.1)		
Female	19	17.0	19	17	38 (33.9)		
**Residential area**						0.242	0.010*
East Tahuna	1	0.9	0	0.0	1 (0.9)		
West Tahuna	6	5.4	15	13.4	21 (17.8)		
Tahuna	1	0.9	1	0.9	2 (1.8)		
Middle Tabukan	1	0.9	1	0.9	2 (1.8)		
Marore	0	0.0	6	5.4	6 (5.4)		
North Tabukan	44	39.3	36	32.1	80 (73.2)		
**Ethnicity**						–0.064	0.501
Sangihe	51	45.5	58	51.8	109 (97.3)		
Jawa	2	1.8	1	0.9	3 (2.7)		
**Religion**						0.172	0.069
Islam	27	24.1	20	17.9	47 (42.0)		
Protestant Christian	26	23.2	39	34.8	65 (58.0)		
**Marriage status**						0.110	0.248
Married	38	33.0	37	33.0	75 (67.0)		
Single	15	13.4	19	17.0	34 (30.3)		
Separated	0	0.0	3	2.7	3 (2.7)		
**Education**						–0.133	0.162
Not attending school	3	2.7	2	1.8	5 (4.5)		
Elementary school	7	6.2	19	17.0	26 (23.2)		
Junior high school	10	9.0	4	3.5	14 (12.5)		
Senior high school	26	23.2	32	28.5	58 (51.7)		
Associate degree	5	4.5	1	0.9	6 (5.4)		
Bachelor's degree	0	0.0	1	0.9	1 (0.9)		
Master's degree	2	1.8	0	0.0	2 (1.8)		
**Occupation**						–0.010	0.914
Unemployed	10	8.9	9	8.0	19 (16.9)		
Working in a private company	6	5.4	6	5.4	12 (10.7)		
Fisherman	1	0.9	6	5.4	7 (6.2)		
Farmer	10	8.9	9	8.0	19 (17.0)		
Labor	3	2.7	7	6.2	10 (9.0)		
Housewife	13	11.6	13	11.6	26 (23.2)		
State civil servant	4	3.6	1	0.9	5 (4.5)		
Others	6	5.4	8	7.1	14 (12.5)		
**Income** (**monthly wage)**						0.042	0.661
< Rp. 971,488	40	35.7	42	37.7	82 (73,3)		
Rp. 971,488–Rp. 3,485,000	9	8.0	13	11.6	22 (19.6)		
> Rp. 3.485.000	4	3.5	4	3.5	8 (7.1)		
**Raw fish consumed**						0.281	0.003*
*Thunnus albacares*	4	3.6	3	2.7	7 (6.3)		
*Hemiramphus brasiliensis*	32	28.6	17	15.2	49 (43.8)		
*Euthynnus affinis*	1	0.9	5	4.5	6 (5.4)		
*Scomberomorus commerson*	2	1.8	5	4.5	7 (6.3)		
*Katsuwonus pelamis*	7	6.3	16	14.3	23 (20.5)		
*Decapterus macrosoma*	4	3.6	5	4.5	9 (8.0)		
*Decapterus kurroides*	1	0.9	3	2.7	4 (3.6)		
*Auxis thazard*	0	0	2	1.8	2 (1.8)		
*Loligo *sp.	1	0.9	1	0.9	2 (1.8)		
*Stolephorus *sp.	0	0	1	0.9	1 (0.9)		
*Centrophorus* sp.	1	0.9	1	0.9	2 (1.8)		
**Knowledge**						–0.064	0.501
Moderate	2	1.8	1	0.9	3 (2.7)		
Poor	51	45.5	58	51.8	109 (97.3)		
**Attitude**						–0.116	0.222
Positive	29	25.9	39	34.8	68 (60.7)		
Negative	24	21.4	20	17.9	44 (39.3)		
**Practice**						0.283	0.003*
Good	8	7.1	24	21.4	32 (28.6)		
Poor	45	40.2	35	31.3	80 (71.4)		

N = Number; *r* = Spearman rank correlation; *Statistically significant at *p *< 0.05.

The final model had a Nagelkerke *R*^2^ value of 0.687, indicating that the independent variables (age, residential area, religion, marital status, education, raw fish consumption, attitudes, and practices) could explain 68.7% of the variation in seropositive anisakiasis cases. The remaining 31.3% were affected by unknown causes.

The study found that the two most important predictors of seropositive anisakiasis were raw *K. pelamis* consumption (*p* = 0.015) and inadequate practices (*p* = 0.001) (Table 2). Other confounding variables included age, residential area, religion, marital status, education, and attitudes.

Among all risk variables, raw *K. pelamis* intake had the greatest odds ratio (OR = 42.910, 95% CI: 2.103–875.391). After controlling for all factors, people who consumed uncooked *K. pelamis* had a 42.91 times higher chance of anisakiasis than those who consumed *T. albacares*. The confidence interval (CI) omitted the null value (1), demonstrating the statistical significance of this connection. These data indicate that *K. pelamis* may be a key vector for *Anisakis* spp. transmission in this community.

Furthermore, poor practices (OR = 17.689) were substantially linked with anisakiasis risk, emphasizing the need to raise public knowledge about safe seafood consumption habits.

## Discussion

Anisakiasis is an important public health concern that has an impact on the economy. Anisakiasis can cause gastric symptoms that appear a few hours after consuming *Anisakis* larvae, including abdominal pain, nausea, vomiting, and diarrhea [19]. Intestinal symptoms occur 2–3 days later, with severe abdominal pain and potentially serious complications such as intestinal obstruction [20]. From an economic perspective, anisakiasis can affect the fishing and seafood industries. If there is an increase in cases, it is feared that this can reduce consumer confidence in seafood products, which can then result in a decrease in demand and economic losses for fishermen, traders, and exporters [21]. In addition, the cost of treatment and health management for this disease increases the economic burden on the health system.

In this study, 52.67% of the respondents were seropositive for anisakiasis, which is much higher than the 7% reported in China and 11% in Sidoarjo, Indonesia [8,15]. This difference in prevalence was due to differences in sampling techniques. In China, only 21.6% of the sample consisted of individuals who consumed raw or undercooked seafood, while in Sidoarjo, the sample came from hospital patients with diarrhea. This may not fully represent the general population at risk of anisakiasis. In contrast, our study focused on individuals who regularly consumed raw fish, increasing the likelihood of identifying seropositive cases. Furthermore, regional food patterns, ingested fish species, and parasite distribution may all contribute to the greater prevalence identified in our research.

The finding of this study was the absence of anisakiasis symptoms among the respondents. This may be due to the different immune responses of the respondents. Th2 cells play a critical role in defense against helminth parasites [22]. This difference in immune response can lead to differences in serological test results and clinical manifestations.

Recent research suggests that anisakiasis may occur without clinical symptoms. A Japanese study found that 22.2% of patients with gastric anisakiasis were asymptomatic and that the main risk factors were older age, male sex, and gastric mucosal atrophy. Further analysis indicated that changes in the condition of the gastric mucosa may affect the immune response to Anisakis infection, thereby decreasing the likelihood of symptoms. In addition, asymptomatic cases of anisakiasis have been discovered incidentally through colonoscopy. These findings suggest that Anisakis infection may go undiagnosed in asymptomatic individuals [23].

In some cases, the *Anisakis* infection can also be asymptomatic. This finding was confirmed by a case report of colonic anisakiasis in Japan. The report found no signs of inflammation, granulomatous lesions, or submucosal tumors in the colon infected with *Anisakis* larvae [24]. Another report in Japan also mentioned the absence of symptoms in a 79-year-old patient with gastric anisakiasis [25]. This may occur if *Anisakis* larvae are in a location that does not cause significant tissue irritation or damage. Therefore, these individuals are thought to be asymptomatic.

**Table 2.** Multivariate logistic regression analysis of variables associated with anisakiasis.

Nagelkerke *R*^2^	Variable	*p* value	OR	CI 95%
0.687	**Age (years)**			
	17–24	Ref		
	25–34	0.978	0.975	0.155–6.131
	35–44	0.797	1.367	0.126–14.869
	45–54	0.266	5.132	0.287–91.850
	≥ 55	0.105	0.061	0.002–1.797
	**Residential area**			
	East Tahuna	Ref		
	West Tahuna	1.000	0.749	0.000
	Tahuna	1.000	2.319	0.000
	Middle Tabukan	1.000	0.001	0.000
	Marore	0.999	1174987126608842500.000	0.000
	North Tabukan	1.000	2.663	0.000
	**Religion**	0.079	4.929	0.830–29.268
	**Marriage status**			
	Married	Ref		
	Single	0.377	2.105	0.403–10.990
	Separated	0.999	2308155534	0.000
	**Education**			
	Not attending school	Ref		
	Elementary school	0.908	1.288	0.018–94.028
	Junior high school	0.132	0.016	0.000–3.462
	Senior high school	0.497	0.171	0.001–27.984
	Associate degree	0.999	0.000	0.000
	Bachelor's degree	0.999	35384492047236976.000	0.000
	Master's degree	0.999	0.000	0.000
	**Raw fish consumed**			
	*Thunnus albacares*	Ref		
	*Hemiramphus brasiliensis*	0.955	1.082	0.069–16.875
	*Euthynnus affinis*	0.323	6.607	0.156–279.323
	*Scomberomorus commerson*	0.999	0.000	0.000
	*Katsuwonus pelamis*	0.015*	42.910	2.103–875.391
	*Decapterus macrosoma*	0.538	3.451	0.067–177.986
	*Decapterus kurroides*	0.360	6.056	0.128–287.233
	*Auxis thazard*	0.999	1198043395705940740.000	0.000
	*Loligo sp.*	1.000	0.000	0.000
	*Stolephorus sp.*	1.000	18084185547.030	0.000
	*Centrophorus sp.*	0.159	23.700	0.283–1948.089
	**Attitude**	0.315	0.502	0.131–1.927
	**Practice**	0.001*	17.689	3.102–100.889

OR = Odds ratio; CI 95% = 95% Confidence interval; *Statistically significant at *p *< 0.05

Another suspicion for asymptomatic patients in our study was the IgG serology test. This test reflects past exposure to the infection so that antibodies specific to the pathogen are formed [26]. This suggests that IgG antibodies against *Anisakis* can remain in the body even if the infection is inactive or asymptomatic. One study explained that there are variations in the immune response to *Anisakis* antigens among different patients and that factors such as sampling time and phase of infection may affect the test results [27].

Immunoglobulin M (IgM) and IgG antibody levels play an important role in the immune response to *Anisakis* infection. Specific IgM antibody levels increased from the sixth day, peaked on the day after infection, and decreased thereafter. Serum levels of IgG antibodies increase from the sixth day, peak on the twenty-sixth day after infection, and decline gradually thereafter [28]. IgG antibodies function to mark and neutralize *Anisakis* antigens and provide long-term protection with immunological memory, allowing the body to respond more quickly if exposed again in the future. IgG levels are known to decrease and potentially disappear when the immune system no longer responds to parasitic antigens. Although specific evidence for this phenomenon in anisakiasis is currently lacking, studies on other parasitic infections, such as bancroftian filariasis, have shown that subjects who recover from microfilaremia experience a marked decrease in IgG levels compared to those with persistent infection. These observations suggest that the same mechanism may occur in anisakiasis; therefore, further research is needed to confirm this possibility [29].

*Anisakis* infection is often associated with increased numbers of neutrophils or eosinophils in the blood as well as tissue damage due to interactions between the immune system and substances secreted by the larvae. Eosinophils exhibit chemotaxis toward *Anisakis* extracts, whereas neutrophils can be triggered by tissue damage. In biopsies of anisakiasis patients, an increase in eosinophil basic protein, nitric oxide synthase, and eosinophil cationic protein in the serum was detected in the first 72 h of infection. These substances have the potential to kill larvae but can also cause localized tissue damage [30].

When *Anisakis* sp. worms enter the gastrointestinal tract, the host immune response is activated. Expulsion of adult worms in the gut involves mast cells, goblet cells, and non-hematopoietic cells, such as smooth muscle cells and epithelial cells, which are regulated by Th2 cytokines create an intestinal environment unfavorable for worm survival. If worm larvae migrate through the lungs or skin, the same response occurs at these sites. Damage to epithelial and epidermal cells releases Th2-inducing cytokines such as IL-33 and thymic stromal lymphopoietin. ATP released from damaged cells promotes Th2 responses in the lungs. Th2 cytokines, which are similar to those involved in allergic diseases, cause symptoms such as diarrhea, vomiting, hives, angioedema, urticaria, anaphylaxis, and allergic symptoms such as asthma, rhinoconjunctivitis, and atopic dermatitis, which are triggered by mast cell mediators [30].

A particular problem with serological testing for nematodes that needs to be addressed is the possibility of cross-reactivity. The diagnostic serology kit we used was highly sensitive (97%) and specific (97%) for anisakiasis. However, in our diagnostic performance, there was a possibility of cross-reactivity (19%) with other parasitic infections (toxocariasis, filariosis, and strongyloidosis). A limitation of our study is that we did not validate the cross-check between *Anisakis* infection and other parasitic infections, which may have affected the diagnostic results and data interpretation. Despite this possibility, the results obtained provide a relevant picture of the prevalence of anisakiasis in the study population. Further research is needed to validate these results and investigate cross-reactions of anisakiasis with other parasitic infections. This potential cross-reactivity may lead to an overestimation of the prevalence of anisakiasis due to false-positive results. This has implications for the validity of the study, as individuals who are not infected with *Anisakis* spp., it may be seropositive. Therefore, additional diagnostic methods or combinations with molecular techniques are required to improve the detection accuracy and reduce the risk of misinterpretation of results.

Bivariate test results indicated that respondents’ residential areas had a statistically significant correlation with seropositive anisakiasis. However, the weak correlation strength indicates that while location may be associated with anisakiasis risk, it is not a dominant factor. This suggests that other factors, such as individual consumption habits, hygiene practices, and knowledge of parasite risk, may play a more substantial role. Residential areas reflect social and environmental influences, including cultural dietary norms and communal eating behaviors, which can shape food choices [31]. People tend to conform to local dietary habits as social adaptation is often beneficial [32]. However, since anisakiasis is primarily transmitted through raw fish consumption, the weak correlation suggests that direct dietary behaviors and food-handling practices have a greater impact on infection risk than location alone.

The distribution of anisakiasis seropositivity was higher in coastal areas far from the city center. We suspect that this is related to limited food choices, which makes locals more likely to consume raw or undercooked fish. This consumption pattern is influenced by the availability of local food, and the lack of education about the risk of anisakiasis increases the seropositivity rate.

The results of the bivariate study, which were further analyzed using multivariate tests, revealed that raw *K. pelamis* consumption was a risk factor that was significantly correlated with seropositive anisakiasis. Anisakiasis, a gastrointestinal disease caused by parasitic nematodes, poses a risk through the consumption of raw or undercooked fish, including skipjacks (*K. pelamis*). In Tokyo, Japan, a surge in anisakiasis cases associated with skipjack was observed in 2018, and *Anisakis simplex* was identified as the main causative species [33,34]. The ventral muscle of the skipjack was found to be particularly susceptible to *Anisakis* infection, suggesting that avoiding raw consumption of this part may help prevent anisakiasis [34].

In Indonesia, some fish species are commonly consumed raw, especially in traditional dishes such as Kinilo. The most commonly consumed fish is *K. pelamis*. Studies in Indonesia have also reported *Anisakis* infection in Skipjack, with a prevalence rate of 26.66% in Kupang City [35] and the identification of *Anisakis* *typica* in the Sawu Sea [36]. However, reports from the Marore Islands indicate that the parasite found in *K. pelamis* is *Rhadinorhynchus* sp., with a prevalence of 100% [37]. This finding illustrates the variation in parasite species in skipjacks in different regions, which continues to emphasize the potential risk of anisakiasis associated with the consumption of this fish species.

The results also indicated that the practice variable was associated with anisakiasis risk (*OR* = 17.689). The lack of attention to the presence of *Anisakis* parasites during fish processing and the inability to clean them properly could be potential risk factors for anisakiasis.

Raw seafood consumption behaviors that pose a high risk of anisakiasis can be prevented and controlled through proper storage methods such as freezing [38]. Studies have shown that proper storage temperature and time can affect the postmortem motility of *A. simplex* larvae in fish, and maintaining the temperature below 2°C can prevent larval migration into the meat during storage, handling, and transport [39]. To kill the larvae, it is recommended to freeze fish at −20°C for 48 to 72 h or −35°C for 15 h [1]. In addition, cooking fish at temperatures above 60°C effectively prevents infection because larvae cannot survive at these temperatures [40]. Another effective method is salting, where studies show that larvae experience reduced survival after being immersed in a 15% or 20% salt solution for 2 days [41]. The salting process can damage the larval cuticle and digestive tract, thereby modifying the permeability of the cell membrane and leading to leakage of cell contents [42]. Therefore, the combination of freezing before salting may increase the effectiveness of killing the *Anisakis* larvae.

Eating Kinilo is an existing habit that is difficult to change or prohibit. This habit can be combined with garlic consumption. This was revealed in a study using mice as test animals that had been inoculated with *Anisakis* larvae and administered garlic oil. The results of this study provided significant protection to infected mice, as they showed normal histological examination results for the liver and kidneys. Garlic has health benefits, including boosting the immune system, reducing inflammation, fighting oxidation, and killing microbes [43].

Given the significance of this strategy in anisakiasis control, the rules that might be enacted include strict regulations on freezing fish before sale, particularly for raw eating. Public health initiatives are also required to promote awareness of the dangers of anisakiasis and to teach appropriate seafood handling. Furthermore, engagement with the fishing sector to standardize storage and distribution practices can be a significant step toward lowering the risk of infection in the community.

However, assessing the actual impact of these preventive measures requires reliable data that can be influenced by the sampling method used. The primary problem in this study was the use of a volunteer sampling strategy, which may have resulted in selection bias. Willing participants may be more health conscious or have prior experience with raw fish intake, rendering the results less representative of the public as a whole. Furthermore, people who ingest raw fish regularly but do not exhibit symptoms may be less motivated to participate, influencing the assessment of anisakiasis prevalence. Therefore, future studies should adopt a random sampling strategy to make the results more representative.

## Conclusion

The study concludes that this is the first report on the seroprevalence of anisakiasis in the Sangihe Islands. It shows how important it is to take action to fix the health problems caused by eating raw seafood. A holistic approach based on the concept of “One Health,” which includes communication, coordination, and collaboration across sectors, is needed to reduce the risk factors for anisakiasis. These interventions should include individual behavior changes, supportive policies, health education, and prevention programs at the community and population levels. We hope that these integrated measures can effectively combat anisakiasis through prevention and systemic improvements.

## List of abbreviations

sp, single species within a genus; spp, multiple species within a genus; g, the relative centrifugal force; °C, degree Celsius; min, minutes; nm, nanometer; *r*, Spearman’s rank correlation coefficient; OR, odds ratio; Th2, T helper type 2; IgG, immunoglobulin G; IgM, immunoglobulin M; h, hour/hours; IL-33, interleukin-33; TSLP, thymic stromal lymphopoietin; ATP, adenosine triphosphate.
